# Association between TLR4 and PTEN Involved in LPS-TLR4 Signaling Response

**DOI:** 10.1155/2016/6083178

**Published:** 2016-08-02

**Authors:** Huahua Yin, Yan Tan, Xiaofeng Wu, Hong Yan, Feng Liu, Yuanzhang Yao, Jianxin Jiang, Qi Wan, Lei Li

**Affiliations:** ^1^Department of Infection & Immunity, Research Institute of Surgery, Daping Hospital, Third Military Medical University, 10 Changjiang Zhilu, Daping, Chongqing 400042, China; ^2^Department of Trauma, Daping Hospital, Third Military Medical University, 10 Changjiang Zhilu, Daping, Chongqing 400042, China; ^3^State Key Laboratory of Trauma, Burns and Combined Injury, Research Institute of Surgery, Daping Hospital, Third Military Medical University, 10 Changjiang Zhilu, Daping, Chongqing 400042, China; ^4^Toronto Western Research Institute, University Health Network, University of Toronto, 399 Bathurst Street, Toronto, ON, Canada M5T 2S8

## Abstract

In this study, we explored the potential mechanisms of how PTEN regulating LPS induced TLR4 signaling pathway. The initial findings from ELISA demonstrate that PTEN influences TNF-*α* secretion by its lipid phosphatase activity. Subsequently, western blot, immunoprecipitation assay, and immunofluorescence were performed to explore the activation process of PTEN by stimulation with LPS. As early as 20 minutes after LPS stimulation, reduced phosphorylation of PTEN was found obviously. Accordingly, the whole cell-scattered PTEN translocated towards the cell membrane 20 minutes after stimulating with LPS. Moreover, the weak physical association between PTEN and TLR4 in resting RAW264.7 cells increased gradually after the stimulation of LPS. Furthermore, our study showed PTEN decreased LPS-induced Akt activity and upregulated NF-*κ*B-dependent gene transcription, identifying indirectly that the PTEN could regulate the activation of NF-*κ*B by its downstream Akt kinase. In summary, our study illustrates the potential signal transduction process of PTEN while stimulated by LPS: by increasing the association of TLR4, PTEN recruits to its phosphoinositide substrate PI(3,4,5)P3 located on the cell membrane and exerts its dephosphorylated function and subsequently depresses the activity of downstream molecule Akt and results in activation of NF-*κ*B, followed by the secretion of inflammatory mediators TNF-*α*.

## 1. Introduction

Current wisdom implies that after severe injury or infectious challenge some patients respond by overexpressing inflammatory mediators which results in a systemic inflammatory response culminating in severe shock, multiorgan failure, and even death [1–4].

As a major component of the outer membrane of Gram-negative bacteria, lipopolysaccharide (LPS) is the principal initiator of sepsis or septic shock. Considerable research has focused on the molecular mechanism of LPS-induced sepsis, especially the cellular signaling of activation of toll-like receptor 4 (TLR4) in the past few years. TLR4 signaling has been divided into MyD88-dependent and MyD88-independent pathways. MyD88-dependent pathway is responsible for proinflammatory cytokine expression while the other one mediates the induction of Type 1 interferons and interferon-inducible genes. Recent studies have shown that TLR4 signaling can be regulated at multiple levels by many regulators which target different key molecules in the TLR4 signaling [5–8]. The improper regulation of LPS/TRL4 signaling has the potential to induce massive inflammation and cause acute sepsis; therefore, it is important to further explore and evaluate the molecules which might be involved in the regulation of TLR4 signaling.

PTEN (phosphatase and tensin homolog deleted on chromosome ten) was cloned as a tumor suppressor for gliomas in 1997 [9] and has been identified to be involved in most tumorigenesis. PTEN functions as a lipid phosphatase and as a protein tyrosine phosphatase. As a lipid phosphatase, PTEN antagonizes PI3K (phosphatidylinositol-3-kinase)/Akt signaling by dephosphorylating phosphatidylInositol (3,4,5)-trisphosphate (PIP3) at position 3 and is involved in various cellular processes including survival, proliferation, energy metabolism, and cellular architecture [10]. Furthermore, the protein phosphatase activity of PTEN influences cell motility, invasion, and migration [11].

Recent studies illustrate that PTEN might play a critical role in inflammatory response. Zhu and his colleagues state that PTEN depleted neutrophils caused more serious inflammatory response compared with wild-type neutrophils [12]. Agrawal et al.'s research demonstrates that increased expression of PTEN in elderly individuals resulted in significant increase of LPS-induced secretion of TNF-*α* and IL-6 [13]. Cao's groups indicate that PTEN is an intracellular positive regulator of LPS-TLR4 signaling and mediator of macrophage inflammatory responses. However, there is a dearth of literature regarding how PTEN regulated LPS-induced signal transduction and its mechanism. The aim of this study is to identify the impact of LPS on PTEN expression and activation, explore the relationship between PTEN and TLR4, and clarify the role of PTEN in regulating LPS-induced signal transduction and the inner cellular signal transduction of PTEN after LPS stimulation.

## 2. Materials and Methods

### 2.1. Reagents

Wild-type PTEN, G129E-PTEN (lipid phosphatase activity-disrupted mutant PTEN), C124A-PTEN (lipid and protein phosphatases activity-disrupted mutant PTEN), and CMV (control empty vector) were provided by Professor Qi Wan (Toronto Western Research Institute, University Health Network, University of Toronto), and all the plasmids were identified by sequencing. LPS (*Escherichia coli* serotype 0111:B4) was purchased from Sigma-Aldrich, RPMI1640 medium was obtained from Gibico, USA, and bovine fetal serum was obtained from Hyclone Co. Phospho-PTEN Ab, PTEN Ab, Phospho-Akt Ab, and Akt Ab were purchased from Cell Signaling Technology Inc., and TLR4(L-14):sc-16240 was purchased from Santa Cruz Biotechnology. Monoclonal Anti-*β*-Actin was purchased from Sigma. Bisperoxovanadium [bpV(Pic)] was purchased from ALEXIS, Lipofectamine 2000 transfection reagent was obtained from Invitrogen, USA, and mouse TNF-*α* ELISA kit was purchased from JingMei Company, China. Dual-Luciferase Reporter Assay System was purchased from Promega, USA. Labeled Donkey Anti-Goat IgG antibody was from Alexa Fluor 555 and Donkey Anti-Rabbit IgG antibody was from Alexa Fluor 488.

### 2.2. Cell Lines and Culture Condition

RAW264.7 cells, originally acquired from the American Type Culture Collection, were provided by Professor Yuzhang Wu (Department of Immunology, Third Military Medical University, Chongqing, China). Cells were cultured in RPMI1640 medium supplemented with 10% FBS, and serum was removed overnight before LPS stimulation.

### 2.3. SDS-PAGE and Immunoblotting

RAW264.7 cells were treated with LPS (1 *μ*g/mL) for different lengths of time (5, 10, 20, 30, 45, 60, and 120 min), cell lysate was harvested, and the level of phosphorylated PTEN, as well as total PTEN protein, was detected by western blot.

In the other experiment, RAW264.7 cells were seeded in culture flask, cells were left untreated or treated with the PTEN inhibitor for 1 hour, followed by stimulation of LPS (1 *μ*g/mL) for 0, 10, 20, 30, 45, and 60 min, cell lysate was harvested, and p-Akt and the total protein levels were detected accordingly by western blot.

In a parallel experiment, RAW264.7 cells were seeded in culture flask and transfected next day with 8 *μ*g of PCMV-GFP vector alone or PCMV vectors containing PTEN cDNA, using the Lipofectamine 2000 20 *μ*L (Lipofectamine*™* 2000 protocol). About twenty hours after transfection, culture medium was changed to the serum-free RPMI 1640 for 8–12 hours, cells were treated with LPS (1 *μ*g/mL) for 0, 15, and 30 min, and then cell lysate was harvested. p-Akt and the total protein levels were detected accordingly by western blot.

### 2.4. Immunofluorescence

RAW264.7 cells were plated on Fn-coated cover for slides 12 hours before assay, starved, stimulated (0, 20, 60, and 120 min, 37°C) with LPS 1 *μ*g/mL, and then washed by PBS three times and fixed with 4% paraformaldehyde (20 minutes, 20°C) in PBS. After being washed three times by PBS, cells were then permeabilized with 0.3% Triton X-100 (20 minutes, 20°C) and incubated (1 hour, 4°C) with 5% goat serum. Cells were cultured with a PTEN antibody and a TLR4 antibody overnight (4°C) and then washed by PBS three times, followed by fluorescence conjugated secondary antibody, respectively, for 1 hour (20°C) in a dark environment. After being washed by PBS three times, samples were mounted in Vectashield medium and images were recorded in a confocal microscope (Leica).

### 2.5. Coimmunoprecipitation, Western Blotting, and* In Vitro* Binding Assays

After appropriate periods of cultivation, cells were washed twice with PBS and scraped into lysate buffer containing 1 mM dithiothreitol (DTT), 1 mM phenylmethylsulfonyl fluoride (PMSF), EDTA solution at 10 *μ*L/mL, and Halt*™* protease inhibitor cocktail (Pierce Company, IL, USA) 10 *μ*L/mL. The cells were sonicated for 10 s and then centrifuged for 5 min at 10,000 ×g. Immunoprecipitation and western blot assays were performed as described previously [13]. Primary antibodies were labeled with horseradish peroxidase-conjugated secondary antibodies, and bands were imaged using an ECL detection system (Amersham Biosciences, Baie D'urfe, Quebec, Canada).

### 2.6. Transit Transfection of RAW264.7 Cells and Luciferase Assay

RAW264.7 cells were transfected by Lipofectamine 2000 with 0.8 *μ*g wild-type PTEN, C124A-PTEN, G129E-PTEN, control empty plasmid (CMV), 0.2 *μ*g NF-*κ*B-Luc plasmid, and 4 ng of pRL-CMV-Renilla-luciferase plasmid. Twenty-four hours later, the transfected cells were left untreated or treated with LPS (1 *μ*g/mL) for 6 hours. In the other experiment, RAW264.7 cells were transfected by Lipofectamine 2000 with 0.8 *μ*g NF-*κ*B-Luc plasmid and 16 ng of pRL-CMV-Renilla-luciferase plasmid. Twenty-four hours later, the transfected cells were cultured with or without PTEN inhibitor for 1 hour, followed by the stimulation of LPS (1 *μ*g/mL) for another 6 hours. Luciferase activities were measured using Dual-Luciferase Reporter Assay System according to the manufactures' instructions. Data are presented as the relative fold increase in LPS stimulated sample readout over nonstimulated sample readout and are expressed as the mean ± SD.

### 2.7. ELISA Determination of Cytokine Production

RAW264.7 cells were transfected with wild-type PTEN, mutant PTEN, or control empty plasmid (CMV) and cultured for 24 hours and then stimulated with 1 *μ*g/mL LPS for another 6 hours. Concentrations of TNF-*α* in the supernatants were measured by ELISA. Data are presented as the relative fold increase in LPS stimulated sample readout over nonstimulated sample readout and are expressed as the mean ± SD. In another experiment, RAW264.7 cells were treated with or without PTEN inhibitor for 1 hour, followed by the stimulation of LPS for 6 hours; concentrations of TNF-*α* in the supernatants were measured by ELISA readout and are expressed as the mean ± SD.

### 2.8. Statistical Analysis

The ECL signal was quantified with Quantity One densitometry program (Bio-Rad). Phosphorylation data were normalized and expressed as the fold change from nonstimulated samples. All data are expressed as the mean ± SD derived from at least three independent experiments. The *t*-test and ANOVA analysis were used when appropriate to examine the statistical significance of the differences between groups of data. Statistical significance was placed at *P* < 0.05.

## 3. Results 

### 3.1. PTEN Promoted LPS-Induced TNF-*α* Secretion in RAW264.7 Macrophages

**Figure 1:**
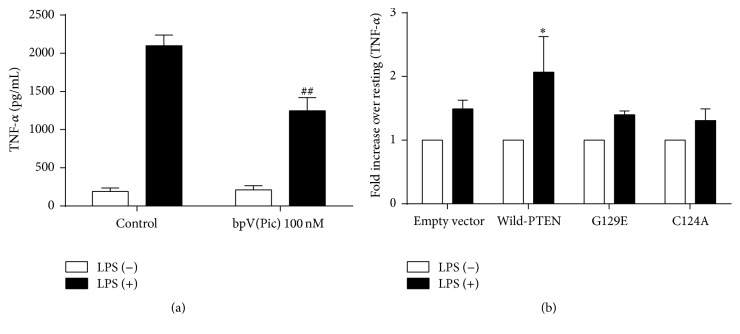
PTEN promoted LPS-induced TNF-*α* secretion in RAW264.7 macrophages (*n* = 7 (a), *n* = 9 (b)). (a) RAW264.7 cells were treated with or without PTEN inhibitor Bisperoxovanadium [bpV(Pic)] 100 nM for 1 hour, followed by the stimulation of 1 *μ*g/mL LPS for 6 hours; concentrations of TNF-*α* in the supernatants were measured by ELISA. Macrophages treated with PTEN inhibitor produced significantly lower levels of TNF-*α* protein in comparison to macrophages without PTEN inhibitor (*P* < 0.01). (b) RAW264.7 cells were transfected with CMV (control empty vector), wild-type PTEN, G129E-PTEN (lipid phosphatase activity-disrupted mutant PTEN), and C124A-PTEN (lipid and protein phosphatases activity-disrupted mutant PTEN), cultured for 24 hours, and then stimulated with 1 *μ*g/mL LPS for another 6 hours. The concentrations of TNF-*α* in the supernatants were measured by ELISA. Data are presented as the relative fold increase in LPS stimulated sample readout over nonstimulated sample readout and are expressed as the mean ± SD. Overexpression of wild-type PTEN enhanced LPS-induced TNF-*α* production in macrophages significantly (*P* < 0.05). However, overexpression of mutant PTEN with lipid phosphatase activity disruption and mutant PTEN with both lipid and protein phosphatases activity disruption did not show the difference with the empty plasmid control group (*P* > 0.05). ^##^ *P* < 0.01 versus bpV(Pic) (−) group; ^*∗*^ *P* < 0.05 versus empty vector group.

In order to identify the role of PTEN in LPS-induced proinflammatory cytokine secretion, the level of TNF-*α* secretion was tested by either using specific antagonist of PTEN to inhibit PTEN function or overexpressing PTEN protein to enhance PTEN function (Figure 1). In comparison to macrophages without PTEN inhibitor, macrophages treated with PTEN inhibitor produced significantly lower levels of TNF-*α* protein (*P* < 0.01) (Figure 1(a)). In addition, overexpression of wild-type PTEN enhanced LPS-induced TNF-*α* production in macrophages (*P* < 0.05). However, overexpression of mutant PTEN with lipid phosphatase activity disruption and mutant PTEN with both lipid and protein phosphatases activity disruption did not show the difference with the empty plasmid control group (*P* > 0.05) (Figure 1(b)). These data suggested that PTEN is involved in LPS stimulating signaling pathway and influences secretion function of macrophages.

### 3.2. PTEN Was Activated and Translocated to Cell Membrane after Stimulation with LPS and at the Same Time Physical Association between PTEN and TLR4 Increased

**Figure 2:**
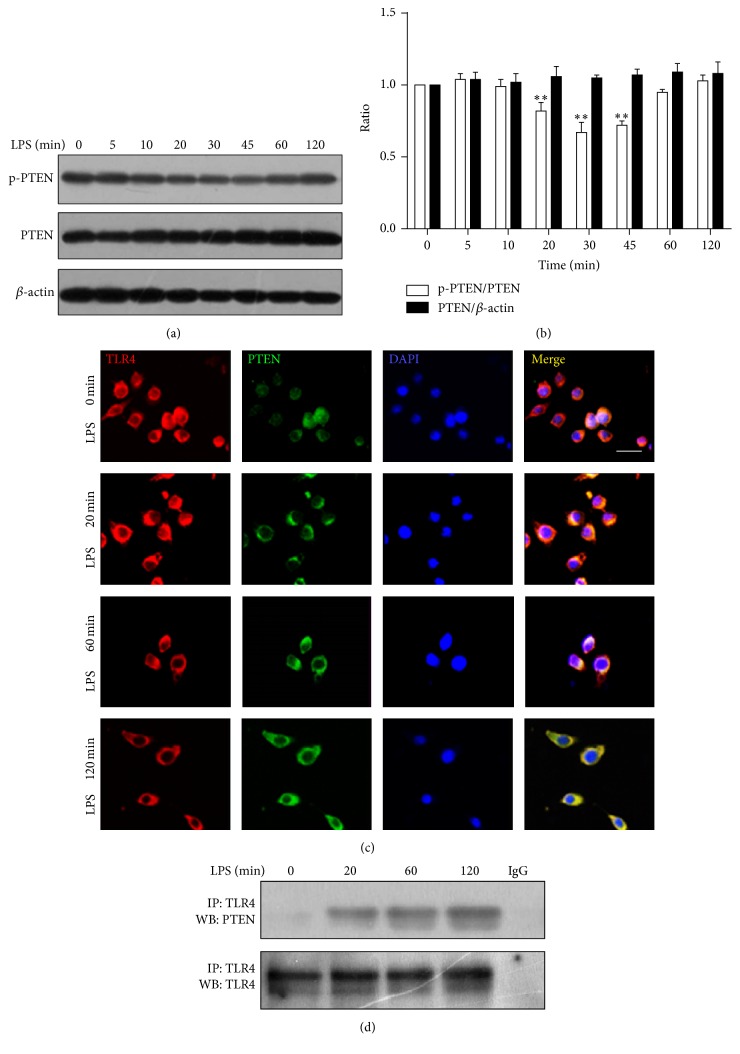
PTEN was activated and translocated to cell membrane after stimulation with LPS. At the same time, physical association between PTEN and TLR4 increased. (a) Phosphorylation levels of PTEN were reduced at 20, 30, and 45 minutes stimulated with LPS at 1 *μ*g/mL. (b) The interaction between PTEN (red) and TLR4 (green), the PTEN in RAW264.7 cytoplasm redistributed towards cell membrane following the LPS stimulation. Scale bar = 15 *μ*m. (c) Cell lysis was immunoprecipitated by TLR4 antibody, and western blot was probed by PTEN antibody. Goat IgG is worked as control. LPS 0 stands for without LPS treatment. The physical associations between PTEN and TLR4 were increased gradually and reached to their peak at 120 minutes after LPS stimulation. Translocation of PTEN in RAW264.7 cells was immunofluorescently stained at 0, 20, 60, and 120 minutes stimulated with LPS at 1 *μ*g/mL by PTEN and TLR4 antibody. ^*∗∗*^ *P* < 0.01 versus LPS 0 min group.

Since functional PTEN often presents in dephosphorylation condition [14], we investigated the phosphorylation level of PTEN in RAW264.7 macrophage stimulation with LPS (0111:B4). As early as 20 minutes, reduced phosphorylation level of PTEN was found in macrophages (Figure 2(a)). Consistently, data from immunofluorescence (Figure 2(c)) identified that PTEN scattered in the whole cell before LPS stimulation, and it translocated towards the cell membrane 20 minutes later after LPS stimulation, merged with TLR4, and became more obvious in 120 min. Interestingly, our finding (Figure 2(d)) showed that that a small amount of PTEN physically associated with TLR4, and the association increased markedly after the stimulation of LPS for 20 min and then reached a peak in 120 min, supporting the results from the immunofluorescence. In summary, these findings showed that PTEN was activated by LPS then targeted to its cell membrane subtracts. In addition, interaction of PTEN and TLR4 may contribute to the process of activation.

### 3.3. PTEN Decreased LPS-Induced Akt Activity

**Figure 3:**
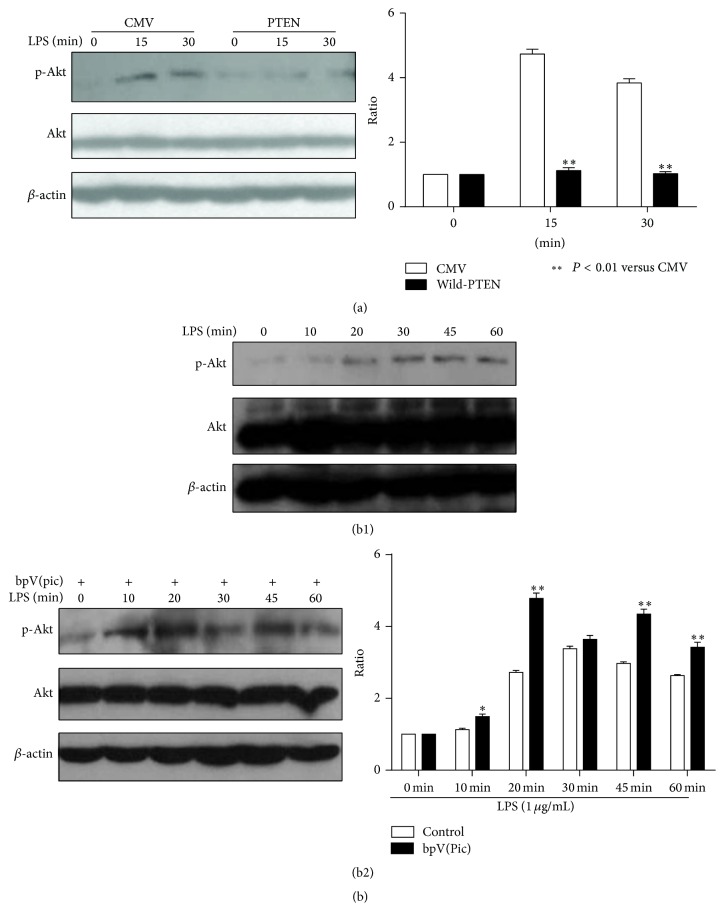
PTEN decreased LPS-induced Akt activity. (a) RAW264.7 cells were transfected with 8 *μ*g of CMV or wild-PTEN with Lipofectamine 2000. About twenty hours after transfection, culture medium was changed to the serum-free RPMI 1640 for another 8–12 hours, cells were treated with LPS (1 *μ*g/mL) for 0, 15, and 30 min, and then cell lysate was harvested. p-Akt and the total protein levels were detected accordingly by western blot. (b) RAW264.7 cells were left untreated or treated with the bpV(Pic) 100 nM for 1 hour, followed by stimulation of LPS (1 *μ*g/mL) for 0, 10, 20, 30, 45, and 60 min; then, cell lysate was harvested and p-Akt and the total protein levels were detected accordingly by western blot. Compared with the cells transfected with CMV, LPS-induced phosphorylation of Akt was significantly inhibited in the cells transfected with wild-type PTEN (*P* < 0.01) (a). On the contrary, LPS-induced phosphorylation of Akt was significantly increased in the presence of bpV(Pic) (*P* < 0.05, *P* < 0.01) (b). ^*∗*^ *P* < 0.05; ^*∗∗*^ *P* < 0.01.

To further understand the effect of PTEN on Akt activity after LPS stimulation, cells were cotransfected with PTEN and control empty vector. Phosphorylation of Akt was significantly inhibited in the cells transfected with wild-type PTEN, compared with the cells transfected with empty vector (Figure 3(a)). On the contrary, LPS-induced phosphorylation of Akt was significantly increased in the presence of PTEN inhibitor compared with the normal condition (Figure 3(b)).

### 3.4. PTEN Upregulates NF-*κ*B-Dependent Gene Transcription in RAW264.7 Macrophages

**Figure 4:**
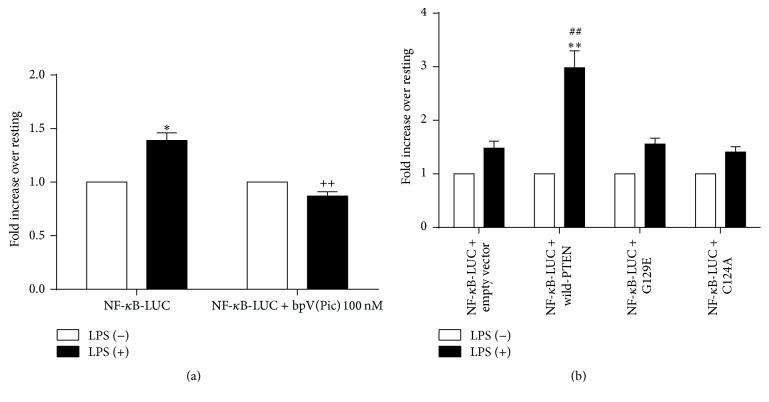
PTEN upregulates NF-*κ*B-dependent gene transcription in RAW264.7 macrophages (*n* = 6 (a), *n* = 9 (b)). (a) RAW264.7 cells were transfected by Lipofectamine 2000 with 0.8 *μ*g NF-*κ*B-Luc plasmid and 16 ng of pRL-CMV-Renilla-luciferase plasmid. Twenty-four hours later, the transfected cells were cultured with or without bpV(Pic) 100 nM for 1 hour, followed by the stimulation of LPS (1 *μ*g/mL) for another 6 hours. (b) RAW264.7 cells were transfected by Lipofectamine 2000 with 0.8 *μ*g CMV/wild-PTEN/G129E-PTEN/C124A-PTEN, 0.2 *μ*g NF-*κ*B-Luc plasmid, and 4 ng of pRL-CMV-Renilla-luciferase plasmid. Twenty-four hours later, the transfected cells were left untreated or treated with LPS (1 *μ*g/mL) for 6 hours. Luciferase activities were measured using Dual-Luciferase Reporter Assay System according to the manufactures' instructions. Data are presented as the relative fold increase in LPS stimulated sample readout over nonstimulated sample readout and are expressed as the mean ± SD. Compared with the bpV(−) group, PTEN inhibitor decreased LPS-induced NF-*κ*B activation significantly (*P* < 0.01) (a). Compared with the empty vector group, overexpressed PTEN enhanced LPS-induced NF-*κ*B activation significantly (*P* < 0.01). No significant effect was found in mutant PTEN groups and empty vector group (b). ^*∗*^ *P* < 0.05 and ^*∗∗*^ *P* < 0.01 versus LPS (−) group. ^++^ *P* < 0.01 versus bpv(Pic) (−) group. ^##^ *P* < 0.01 versus empty vector group.

Compared with the bpV(−) group, PTEN inhibitor decreased LPS-induced NF-*κ*B activation significantly (*P* < 0.01) (Figure 4(a)). Compared with the empty vector group, overexpressed PTEN enhanced LPS-induced NF-*κ*B activation significantly (*P* < 0.01). No significant effect was found in mutant PTEN groups and empty vector group (Figure 4(b)).

## 4. Discussion

During the past twenty years, clinical physicians and researchers have paid more attention to the mechanism of LPS-TLR4 inducing overinflammatory insults on the body. It has been known that cell signaling of the event is involved in two classic pathways, including MyD88-dependent and MyD88-independent pathways [15]. Since the macrophage, as the main innate immune cell, can secrete more than 100 proteins; and the physical functions of macrophage are related to many cytoplasm signal networks [16, 17]. It is suggested that there might be some other cell signaling proteins involved in the LPS-TLR4 inducing inflammatory response except for those in the two conventional pathways. Recently, literatures show that PTEN may play certain roles during the inflammatory reaction [12, 13, 18]; however, its mechanism still remains unclear. There is a paucity of evidence regarding how PTEN is activated by LPS stimulation; the role of TLR4 receptor in PTEN activation; and how PTEN influences LPS-induced cell signal transduction in macrophages. To fill this gap, our study explored the activation process of PTEN by stimulating with LPS and the interaction of PTEN and TLR4 receptor and further identified the function of PTEN in LPS-induced signal transduction.

A primary function of activated macrophages is the rapid and abundant secretion of cytokines [19]. Therefore, the level of TNF-*α* secretion was tested to identify whether PTEN has a role in macrophage secretion function after LPS stimulation. Our finding showed that PTEN promoted the secretion of TNF-*α* after LPS stimulation, supporting the previous studies that PTEN plays a curial role in the LPS-induced inflammatory cytokine secretion of macrophage [20]. Due to PTEN exerting its phosphorylation function by the binding of lipid and protein partners through its two structural domains of phosphatase and C2 which binds lipid domain [14], our study explored which phosphatase took responsibility for the action of PTEN on TNF-*α* production. Findings showed that overexpression of wild-type PTEN enhanced LPS-induced TNF-*α* production in RAW264.7 cells stimulated by LPS; however, LPS-induced production of TNF-*α* was much lower in RAW264.7 cells treated by overexpression of lipid phosphatase activity-disrupted mutant PTEN plasmid and lipid and protein dual phosphatases activity-disrupted mutant PTEN plasmid compared with wild-PTEN plasmid. Furthermore, the production of TNF-*α* between these two disrupted mutant PTEN plasmid groups did not show any significant changes. Overall, our findings suggest that PTEN influences TNF-*α* secretion by its lipid phosphatase activity.

In the following experiments, we try to explore how PTEN activates its lipid substrates mainly located on the cell membrane and how this activation impacts the subsequent inner cell and nuclear signal transduction. Findings from western blot showed that as early as 20 minutes after LPS stimulation, reduced phosphorylation of PTEN was found, suggesting the activation of PTEN by LPS as functional PTEN normally presents in dephosphorylation condition [14]. Accordingly, the findings from immunofluorescence identified that whole cell-scattered PTEN translocated towards the cell membrane 20 minutes after stimulating with LPS, which means PTEN is commencing its function by recruiting to its lipid substrate located dominantly on the inner side of cell membrane [21, 22]. In order to know the potential mechanisms regarding why and how PTEN moves towards the cell membrane, the relationship between the key receptor of LPS signal transduction, TLR4, and PTEN was explored. Interestingly, the findings demonstrated that a small amount of PTEN physically associated with TLR4 in the macrophages, and the association increased markedly after the stimulation of LPS for 20 min and then reached a peak in 120 min. According to our findings, we assumed that after stimulation of LPS, PTEN is activated by interacting with TLR4 and recruits to its lipid substrate on the cell membrane then plays its lipid phosphatase role. In addition, TLR4 is likely to be the potential target facilitating PTEN to attain its substrate on the cell membrane by the increased association with PTEN after the stimulation of LPS. However, unfortunately, we have not identified how those molecules interacted with each other. Therefore, further experiments should be conducted to explore this phenomenon. Even so, our findings are the first to uncover the interaction between TLR4 and PTEN, and the possible role of this interaction in the process of PTEN induced signal transduction.

PTEN is reported as a phosphatidylinositol phosphate (PIP) phosphatase specific for the 3-position of the inositol ring [21]. Although PTEN can dephosphorylate PI(3)P, PI(3,4)P2, and PI(3,4,5)P3* in vitro*, it is likely that PI(3,4,5)P3 is the most important lipid substrate* in vivo* [22]. Research demonstrates that PI(3,4,5)P3 mediates the effects by inducing phosphorylation and activation of the downstream Akt kinase [23]. Again, studies identify that Akt is involved in the cross talk network of LPS signal transduction while it functions as a suppressor of NF-*κ*B. Thus, the impact of PTEN on Akt activation was explored. Consistent with results generated from other studies, our study showed PTEN decreased LPS-induced Akt activity [24–26].

After PTEN was activated by LPS and recruited to its lipid substrates, then influenced phosphorylation of Akt, the function of NF-*κ*B was assessed to identify the nuclear target of PTEN signal transduction as it is regarded the downstream protein of Akt. NF-*κ*B functions as the primary regulator of inflammatory responses and plays a crucial role in the initiation and amplification of inflammatory response. By responding to stimuli such as LPS, NF-*κ*B activation triggers the transcription of mainly proinflammatory target genes such as TNF-*α*. Our findings showed that PTEN upregulated NF-*κ*B-dependent gene transcription, identifying indirectly that the PTEN could regulate the activation of NF-*κ*B by its downstream Akt kinase. This data further supports the previous evidence that Akt negatively regulates NF-*κ*B activation and the expression of inflammatory genes [25]. Similar to the TNF-*α* secretion, overexpressed wild-PTEN also can increase activation of NF-*κ*B significantly; nevertheless, each of those two kinds of mutated PTEN plasmids only elevated slightly NF-*κ*B activation compared with control group. That means only lipid phosphatase activity of PTEN was disrupted, sufficient to abolish PTEN induction on TNF-*α* production as well as activation of NF-*κ*B in RAW264.7 cells treated with LPS. These findings further support the conclusion that the lipid phosphatase function of PTEN leads to induction of NF-*κ*B activation through depressing Akt phosphorylation; therefore, the increased production of TNF-*α* and consequently over inflammatory response even sepsis take place.

In summary, our results suggest that a small number of PTEN physically associate with TLR4 in the normal situation. Stimulation of LPS to RAW264.7 cells could increase the association between PTEN and TLR4, which allows PTEN to recruit to its phosphoinositide substrate PI(3,4,5)P3 located on the cell membrane and exerts its dephosphorylated function and subsequently depresses the activity of downstream molecule Akt, resulting in activation of NF-*κ*B, followed by the secretion of inflammatory mediators TNF-*α*.
